# CYLD alleviates NLRP3 inflammasome-mediated pyroptosis in osteoporosis by deubiquitinating WNK1

**DOI:** 10.1186/s13018-024-04675-2

**Published:** 2024-04-01

**Authors:** Guiyong Jiang, Yu Cai, Duo Cheng, Hao Wang, Geyang Deng, Dayong Xiang

**Affiliations:** 1 Division of Orthopaedics and Traumatology, Department of Orthopaedics, Nanfang Hospital, Southern Medical University, 15th Floor, Surgery Building, Southern Hospital, No.1838 Guangzhou Avenue North, Guangzhou, 510515 Guangdong China; 2 Guangzhou Key Laboratory of Spine Disease Prevention and Treatment, Department of Orthopaedic Surgery, The Third Affiliated Hospital, Guangzhou Medical University, Guangzhou, 510150 Guangdong China

**Keywords:** CYLD, NLRP3, Osteoporosis, Pyroptosis, WNK1

## Abstract

**Background:**

Osteoporosis (OP) is the result of bone mass reduction and bone structure disorder. Bone marrow mesenchymal stem cells (BMSCs) are the main source of osteogenic precursor cells involved in adult bone remodeling. The involvement of the deubiquitinating enzyme CYLD in OP has recently been discovered. However, the detailed role and mechanism of CYLD remain unknown.

**Methods:**

The OP mouse model was established by performing ovariectomy (OVX) on mice. Hematoxylin and eosin staining, Masson and Immunohistochemical staining were used to assess pathologic changes. Real-time quantitative PCR, Western blot, and immunofluorescence were employed to assess the expression levels of CYLD, WNK1, NLRP3 and osteogenesis-related molecules. The binding relationship between CYLD and WNK1 was validated through a co-immunoprecipitation assay. The osteogenic capacity of BMSCs was determined using Alkaline phosphatase (ALP) and alizarin red staining (ARS). Protein ubiquitination was evaluated by a ubiquitination assay.

**Results:**

The levels of both CYLD and WNK1 were decreased in bone tissues and BMSCs of OVX mice. Overexpression of CYLD or WNK1 induced osteogenic differentiation in BMSCs. Additionally, NLRP3 inflammation was activated in OVX mice, but its activation was attenuated upon overexpression of CYLD or WNK1. CYLD was observed to reduce the ubiquitination of WNK1, thereby enhancing its protein stability and leading to the inactivation of NLRP3 inflammation. However, the protective effects of CYLD on osteogenic differentiation and NLRP3 inflammation inactivation were diminished upon silencing of WNK1.

**Conclusion:**

CYLD mitigates NLRP3 inflammasome-triggered pyroptosis in osteoporosis through its deubiquitination of WNK1.

**Supplementary Information:**

The online version contains supplementary material available at 10.1186/s13018-024-04675-2.

## Introduction

Osteoporosis (OP) is a skeletal disease with reduced bone mass and destruction of bone tissue microstructure, resulting in increased fracture risk [1]. As the human society ages, the prevalence of osteoporotic fractures continues to rise annually, imposing a significant burden on society [2, 3]. The fundamental mechanism underlying OP is the disruption in the equilibrium between bone resorption and bone formation [4]. Bone mineral density (BMD) and bone turnover markers (BTMs) are important target parameters to optimize the treatment strategy of OP [5, 6]. Currently, the drugs used in the treatment of OP are mainly bisphosphonates, calcitonins and estrogens, which have serious side effects [7–9]. Therefore, exploration of the specific gene responsible for OP and identification of new therapeutic targets are especially important. Bone marrow mesenchymal stem cells (BMSCs) reside within the bone marrow interstitium with the capacity for self-replication and multi-directional differentiation [10]. Serving as the primary reservoir of osteogenic precursor cells in adult bone remodeling, BMSCs play a vital role in the progression of OP [11]. Therefore, understanding the relevant mechanism of osteogenic differentiation of BMSCs and strengthening the osteogenic differentiation ability of BMSCs will improve the therapeutic effect of OP.

Ubiquitination is a post-translational modification process wherein ubiquitin molecules are covalently attached to protein substrates, resulting in alterations in the stability, localization or activity of the target proteins [12]. Protein ubiquitination is a reversible process, wherein deubiquitinating enzymes can cleave ubiquitin from its protein substrates [13]. Cylindromatosis (CYLD) gene is located on chromosome 16q12-13, and its encoded protein CYLD is considered to be a key regulator of inflammatory response [14]. CYLD exhibits deubiquitinase activity and plays an important role in multiple signaling pathways [15]. CYLD overexpression has been reported to inhibit osteoclast formation by negatively regulating the NF-KB pathway, thereby reducing bone loss [16, 17]. Moreover, CYLD can stimulateosteogenic differentiation by blocking TNF receptor-associated factor 6-mediated polyubiquitination [18]. These clues suggest that CYLD potentially promotes the osteogenic differentiation of BMSCs. However, the precise mechanism and function of CYLD in this context require further investigation.

The protein kinase with no lysine (K) (WNK) kinase family comprises a group of serine/threonine protein kinases that play a crucial role in regulating cellular ion flux, thereby maintaining blood pressure and renal homeostasis [19]. The with no lysine [K] kinase 1 (WNK1) gene is located on chromosome 12p13.33, and its encoded protein WNK1 is a major member of the WNK family [20]. WNK1 is involved in various signaling pathways, such as Wnt signaling pathway [21]. Furthermore, WNK1 has been implicated in regulating fibroblast proliferation, apoptosis and embryonic development [22]. Interestingly, evidence suggests that WNK1 is down-regulated in OP patients with low bone mineral density [23]. Additionally, O-linked N-acetylglucosamine modification, which is associated with early-stage osteoblast differentiation, has been observed in WNK1 as well [24], indicating potential involvement of WNK1 in osteogenic differentiation. Nevertheless, the specific role of WNK1 in OP remains rarely studied.

Inflammasome is a vital molecular structure involved in the body’s defense against pathogens and recognition of endogenous danger signals [25]. Among the various types of inflammasomes, the NOD-like receptor family pyrin domain containing protein 3 (NLRP3) inflammasome has been extensively studied [26]. Upon cellular stimulation, the assembly of NLRP3-ASC adapter protein complex promotes the activation of caspase-1, resulting in the release of pro-inflammatory cytokines such as interleukin-1β (IL-1β) and IL-18, as well as the induction of pyroptosis [27]. Emerging evidence suggests a close association between NLRP3 overexpression and enhanced bone resorption, as well as compromised osteogenesis, with its inflammasome formation leading to osteoblast pyroptosis and dysfunction [28]. Moreover, inhibition of NLRP3 inflammasome formation and pyroptotic activation can facilitate bone formation [29]. Additionally, WNK1 can negatively regulate the activation of NLRP3 by regulating intracellular chloride (Cl-) and potassium (K+) concentrations in the process of renal fibrosis [30]. Therefore, we speculated that WNK1 might promote the osteogenic differentiation of BMSCs by exerting a negative regulatory effect on NLRP3 inflammasome activation.

According to the UbiBrowser V2.0 database, WNK1 protein was identified as a potential target for CYLD-mediated deubiquitination. Therefore, in this study, we hypothesized that CYLD could enhance the stability of WNK1 protein by removing its ubiquitination modification, thereby inactivating NLRP3 inflammasome and promoting the osteogenic differentiation of BMSCs. These findings contribute to a deeper understanding of the underlying mechanisms involved in the pathogenesis of OP, suggesting that CYLD and WNK1 may be potential targets for therapeutic intervention of OP.

## Materials and methods

### OP mouse model

Pathogen-free female C57BL/6J mice (12 weeks) were purchased from Animal Center of the Chinese Academy of Sciences (Shanghai, China). All mice were kept in a carefully controlled environment (12 h light-dark cycle, 25 °C, and 60–70% humidity). Mice were randomly allocated into two groups (*n* = 6), namely the Sham group and the ovariectomized (OVX) group. The OP mouse model was established by ovariectomy as previously described [31]. The mice in OVX group were anesthetized by intraperitoneal injection of sodium pentobarbital (60 mg/kg) and underwent bilateral OVX. Mice of Sham group were subjected to sham operation as control. After 8 weeks, the animals were given euthanasia and satisfaction. Tibial and femoral samples were collected from mice for subsequent experiments. All animal procedures were conducted in accordance with the guidelines and approved by the Animal Ethics Committee of Nanfang Hospital, Southern Medical University.

### Histomorphometric analyses

Mouse femurs were fixed in 4% polyoxymethylene for a duration of 2 days, followed by preservation in 70% ethanol under 4 °C. Image was acquired with the high-resolution micro-CT imaging system (Skyscan 1072, Belgium) under the following conditions, 9-mm isometric resolution, 80 mA and 80 kV. Various bone parameters, including bone mineral density (BMD), bone volume/trabecular volume (BV/TV), trabecular number (TB. N), and trabecular thickness (TB. Th) were measured from the acquired images.

## Hematoxylin and eosin (HE) staining

The dissected mouse femurs were fixed in 4% paraformaldehyde for 24 h, followed by decalcification. Subsequently, the bone specimens were dehydrated using an ascending series of ethanol and embedded in paraffin. Paraffin blocks were then sectioned at a thickness of 5 μm. Sections underwent deparaffinization with xylene and rehydration in a descending series of alcohol. They were first stained with hematoxylin and subsequently with eosin. After undergoing dehydration with gradient ethanol and transparency with xylene, the sections were sealed with resin and observed using an optical microscope (Olympus, Tokyo, Japan).

## Masson staining

The paraffin-embedded bone sections underwent deparaffinization and were subsequently stained with haematoxylin for 8 min, followed by staining with Masson ponceau acid fuchsin solution for another 8 min. The sections were then exposed to aniline blue solution for 8 min and 1% acetic acid for 2 min. After washing, the sections were dehydrated with 95% and anhydrous alcohol, transparentized with xylene, and mounted. Finally, the samples were observed under an Olympus microscope.

### Immunohistochemical staining (IHC)

Bone tissue blocks obtained from mice were fixed in 4% paraformaldehyde and subsequently sectioned. The slides were immersed in 10 mM citrate buffer (pH 7.5) and subjected to antigen retrieval by microwaving at 750 W for 30 min. Following this, the slides were incubated with primary antibodies at 4 °C overnight: NLRP3 (PA5-79740, Invitrogen). Then, the sections were exposed to secondary antibody (ab97080, Abcam) for 1 h. The nuclei were stained with Daminobenzidine (DAB) staining (R&D Systems, Minneapolis, MN, USA). Finally, the slides were mounted and imaged under an Olympus microscope.

### BMSC isolation and culture

Primary BMSCs were isolated from the femurs of mice belonging to the sham and OVX groups (referred to as sham-BMSCs and OVX-BMSCs, respectively) as previously described [31]. Afterwards, cells were incubated in α-MEM (HyClone, USA) supplemented with 10% FBS, (Gibco, Grand Island, NY, USA), 2 mM L-glutamine, 1% Penicillin-Streptomycin (Sigma, St. Louis, MO, USA) and 1% L-glutamine (Invitrogen). The medium was refreshed every three days. BMSCs at passage 3 were utilized for the following experiments. For drug treatment, the transfected cells were treated with 50 ng/ml cycloheximide (CHX, Sigma) for 0, 6, and 12 h.

### Induction of osteogenic differentiation

The isolated BMSCs were cultured in a differentiation-inducing DMEM medium for osteogenesis, which consisted of 10% FBS, 10 nM dexamethasone (Sigma), 10 mM β-glycerophosphate (Sigma), and 50 µM ascorbic acid (Sigma). The cells were maintained in this medium for a period of fourteen days, with the medium being refreshed every three days. Osteogenic differentiation was assessed by ARS and ALP assays as follows.

## Lentivirus construction and cell transfection

Plasmids Flag-CYLD and Myc-WNK1 were constructed and purchased from OBiO Technology (Shanghai, China). Lentiviral plasmids (lv-CYLD, lv-WNK1, lv-sh-WNK1) were purchased from GenePharma (Shanghai, China). The lentiviral vector pLL-3.7 was co-transfected with the packaging plasmid to produce lentivirus particles, which were filtered through 0.45-lm filters, and immediately used for infection. Each lentiviral vector was adopted in BMSCs transfection at the MOI = 30–40. Thereafter, BMSCs (3 × 10^5^ × 10^5^ cells/cm^2^) were inoculated into the 6-well plates. After the cells reached 40-50% density, they were treated with α-MEM (HyClone, USA) without FBS. The original medium was changed after six hours. qRT-PCR was conducted for verifying lentiviral transfection efficiency.

### RNA extraction and quantitative real-time PCR (qRT-PCR)

Total RNA was extracted from cells and tissues using Trizol reagent (Sigma). Subsequently, complementary DNA (cDNA) was reversely transcribed from total RNA with a PrimeScript™ RT Reagent Kit (Takara, Kyoto, Japan). qRT-PCR was conducted using the SYBR Green PCR Kit (Takara) on an ABI 7900HT system (Applied Biosystems, Foster City, CA, USA). The primer sequences used for amplification were provided as follows: CYLD F: 5′-TCCTTGCAGCCTGTTTCCAA-3′, R: 5′-GCCCTGGATGCCTTTCTTCT-3′; WNK1 F: 5′-TCCAACATCCTCAGCAGCAG-3′, R: 5′-GCTGACACTTGAGGCTGACT-3′; RUNX2 F: 5′- AGATGGGACTGTGGTTACCG-3′, R: 5′-GGACCGTCCACTGTCACTTT-3′; OCN F: 5′- GAGACACCACCCCCTGTAAA-3′, R: 5′-ATGACCTTGGCTTCCATGTC-3′; OPN F: 5′- GCTTGGCTTATGGACTGAGG-3′, R: 5′-TGGTTCATCCAGCTGACTTG-3′; GAPDH F: 5′- AGCCCAAGATGCCCTTCAGT-3′, R: 5′-CCGTGTTCCTACCCCCAATG-3′. GAPDH was used as an internal control. The standard 2^−∆∆Ct^ method was utilized to quantify fold change in gene expression.

### Western blot

Cells and tissue samples were prepared using RIPA lysis buffer (Beyotime, Shanghai, China). The protein concentration was detected using the BCA method (Keygen Biotech, Nanjing, China). Equal amounts of proteins were separated on 10% SDS-PAGE gels, followed by electroblotting onto a PVDF membrane (BioRad Laboratories, Inc., Hercules, CA, USA). Following blocking with 5% non-fat milk, the membranes were incubated with primary antibodies at 4 °C overnight: CYLD (ab137524, Abcam), WNK1 (ab35069, Abcam), Runt-related transcription factor 2 (RUNX2, ab236639, Abcam), Osteocalcin (OCN, ab93876, Abcam), Osteopontin (OPN, ab218237, Abcam), NLRP3 (ab263899, 1:1000, Abcam), ASC (ab309497, Abcam), IL-1β (ab205924, Abcam), caspase 1 (ab138483, Abcam). Then, the membranes were washed and further exposed to horseradish peroxidase (HRP) conjugated antibody (#7074, Cell Signaling Technology, Danvers, MA, USA) for 1 h at room temperature. Protein bands were visualized using an enhanced chemiluminescence (Beyotime). The intensity of the bands was quantified using ImageJ software. GAPDH was used as an internal loading control.

### Alizarin red S (ARS) staining

On the 14th day after osteogenic differentiation of BMSCs, mineralization was determined with Alizarin Red S (Sigma) staining. Thereafter, BMSCs were fixed with 4% paraformaldehyde (PFA) for 20 min, and then stained with 1% alizarin red for 5 min. After washing with PBS, images were taken under a microscope (Olympus).

### Alkaline phosphatase (ALP) staining

On the 14th day of osteogenic differentiation induction, BMSCs were fixed with 4% PFA and stained with ALP Detection Kit (Nanjing Jiancheng Biotechnology Institute, Nanjing, China) for 30 min in the dark at room temperature. Thereafter, cells were rinsed with distilled water to stop the color development. The staining pictures were taken under a microscope (Olympus).

### Immunofluorescence staining

BMSCs were fixed with 4% PFA for 30 min, followed by treatment with 0.1% Triton for 15 min. After being washed twice with PBS, the cells were blocked with 5% FBS for 15 min. Anti-NLRP3 antibody (PA5-79740, 1:200, Thermo Fisher Scientific, Waltham, MA, USA) was added to the cells and incubated at 4 °C overnight. After washing, the cells were stained with FITC-labeled goat anti-rabbit IgG (ab6717, 1:1000, Abcam) at 37 °C for 1 h. Following this, the cells were incubated with DAPI for 5 min and washed with PBS. Microscopy of cells was conducted with an Olympus microscope.

### Co-immunoprecipitation (Co-IP)

Flag-CYLD or Myc-WNK1 was transfected into 293T cells using Lipofectamine™ RNAiMAX Transfection Reagent (Invitrogen) for 48 h. Cells were lysed with RIPA buffer (Beyotime, Shanghai, China) and samples were centrifuged to remove cell debris. The supernatant was incubated with anti-Flag antibody (SAB4200071, 1:1000, Sigma) at 4 °C overnight, and precipitated using Protein A/G agarose beads (Roche, Mannheim, Germany) for 30 min. Thereafter, protein A-agarose-antigen-antibody complexes were harvested, followed by rinsing with immunoprecipitation-HAT buffer (1 mL) five times (50 mM Tris-HCl, pH 8.0, 150 mM NaCl, 5 mM EDTA, 0.5% NP-40, and 0.1 mM PMSF). After washing, the eluted proteins were subjected to western blot analysis with anti-Flag (SAB4200071, 1:1000, Sigma) and anti-Myc (13-2500, 1:1000, Thermo Fisher Scientific) antibodies. For endogenous Co-IP experiments, the extracted proteins from OVX BMSCs or normal BMSCs were incubated overnight at 4 °C with a mixture of Protein A/G Plus magnetic beads (Santa Cruz, DBA, Milan, Italy) and antibodies specific to CYLD (ab137524, Abcam), WNK1 (ab35069, Abcam) or IgG (ab2410, Abcam) overnight at 4 °C. Next, the beads were eluted utilizing SDS loading buffer. Finally, protein levels of CYLD or WNK1 were analyzed by Western blot.

### In vitro ubiquitination assay

For ubiquitination assay, lv-CYLD or lv-NC was co-transfected with an ubiquitin-encoding plasmid (HA-UB) into 293T cells stably expressing Myc-WNK1 using Lipofectamine 3000 (Invitrogen) for 48 h. The ubiquitination status of the specified proteins was assessed as previously described [32]. If necessary, 10 μm MG132 (MCE HY-13,259) was added to the cells and incubated for 6 h. Following this, the cells were lysed in 100 ul cell lysis buffer (2% SDS, 150 mM NaCl, 10 mM Tris-HCl, pH 8.0) with 2mM sodium orthovanadate, 50 mM sodium fluoride, and protease inhibitors (Roche). Next, samples were boiled for 10 min, sheared using the sonication device, and then diluted buffer (900 ul, containing 2 mM EDTA, 1% Triton, 150 mM NaCl, 10 mM Tris-HCl, pH 8.0) was added. Each sample was incubated at 4 °C for 30–60 min and rotated, followed by 30-min centrifugation at 20,000 x g, and the supernatant was incubated overnight under 4 °C with anti-c-Myc agarose (A7470, Sigma). Beads were spin down at 5000 x g for 5 min and rinsed with washing buffer (10 mM Tris-HCl, pH 8.0, 1 M NaCl, 1 mM EDTA, 1% NP-40) twice. After aspirating the residual washing buffer, the resin was boiled with 2X SDS loading buffer. The ubiquitination of WNK1 was determined by immunoblotting with anti-HA tag antibody (ab1424, Sigma).

### Statistical analysis

All results were presented as mean ± SD. Student’s t-test was utilized to compare between two groups, while one-way ANOVA was adopted to compare multiple groups. Data analysis was determined using GraphPad Prism version 6. A value of *P* < 0.05 stood for statistical significance. Each assay was carried out thrice or more times.

## Results

### CYLD and WNK1 were decreased in OP mouse model

**Fig. 1:**
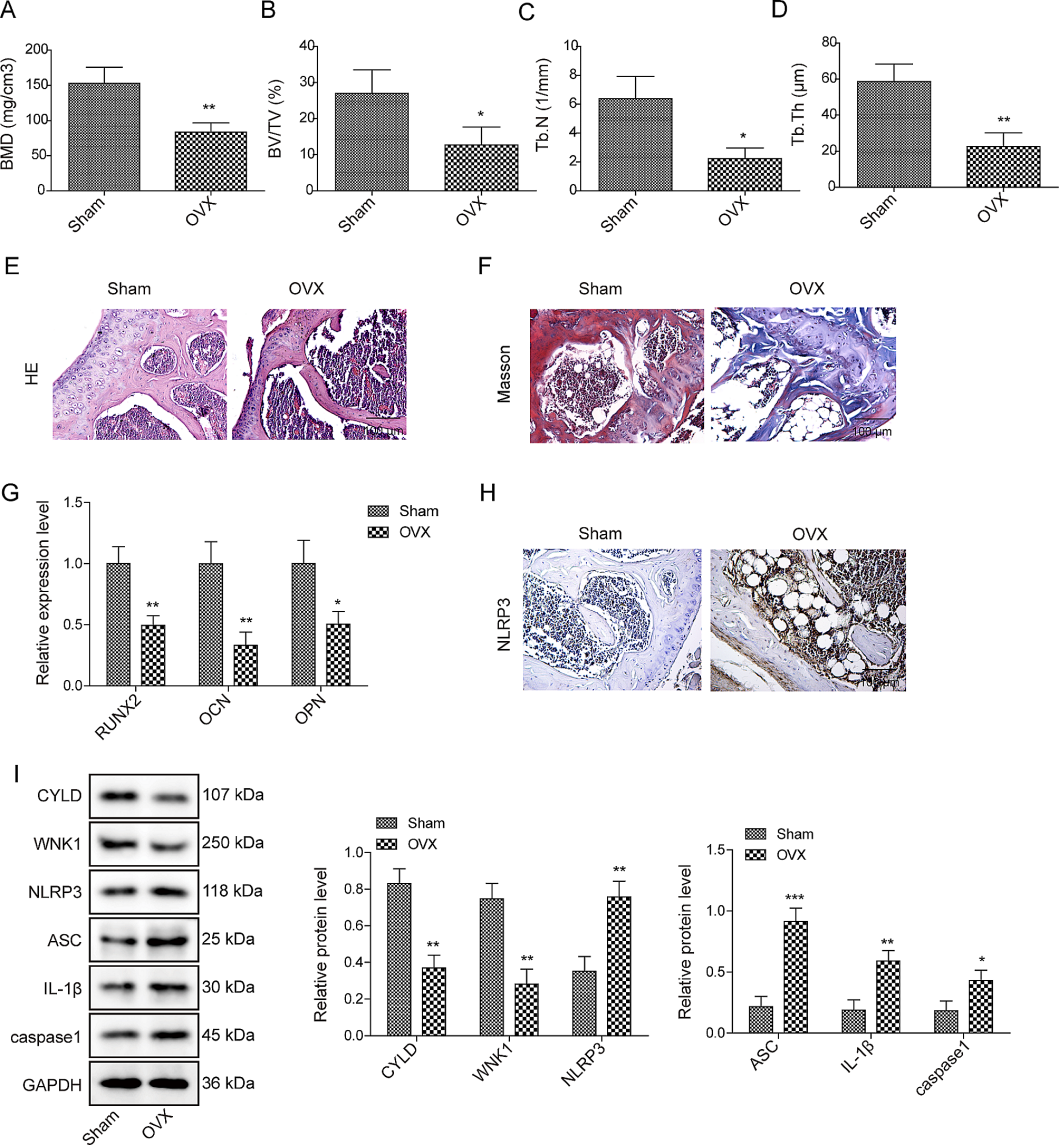
CYLD and WNK1 were decreased in OP mouse model C57BL/6J mice were used for establishing an OP mouse model by OVX method. After experiments, femurs and tibias were collected. **(A-D)** Bone parameters including BMD, BV/TV, Tb.N and Tb.Th were measured. **(E-F)** HE and Masson staining was used to analyze the pathological changes of femurs and tibias. **(G)** qRT-PCR was adopted to measure the expressions of osteogenesis-related molecules (RUNX2, OCN, OPN). **(H)** Immunohistochemical staining was utilized to evaluate the expression of NLRP3. **(I)** Western blot was conducted to detect the levels of CYLD, WNK1, NLRP3, ASC, IL-1β and caspase1. Values are mean ± SD. *P < 0.05, **P < 0.01, ***P < 0.001

Firstly, the OP mouse model was established by OVX method. Micro-CT scanning analysis indicated that compared with the sham group, it could be observed that BMD, BV/TV, Tb.N, and Tb.Th were greatly decreased in OVX mice and the cancellous bone mass in the OVX group was significantly reduced (Fig. 1A and D, Supplementary Fig. 1A). HE and Masson staining revealed that in OVX mice, there was evidence of deteriorated bone microstructure, thinning of the bone cortex, enlarged bone marrow cavity, reduced density or fractured trabecular bone, wider spacing between trabeculae, and decreased collagen content (Fig. 1E-F). Additionally, qRT-PCR results suggested that the mRNA levels of osteogenesis-related molecules (RUNX2, OCN, OPN) were greatly decreased in bone tissues of OVX mice (Fig. 1G). Notably, immunohistochemical assay demonstrated that NLRP3 expression was ascended in OVX mice (Fig. 1H). Similarly, western blot also illustrated that the expressions of CYLD and WNK1 were decreased, but the expressions of NLRP3, ASC, IL-1β and caspase 1 were increased in OVX mice compared to the sham mice (Fig. 1I).

### Overexpression of CYLD or WNK1 promoted osteogenic differentiation of OVX-BMSCs

**Fig. 2:**
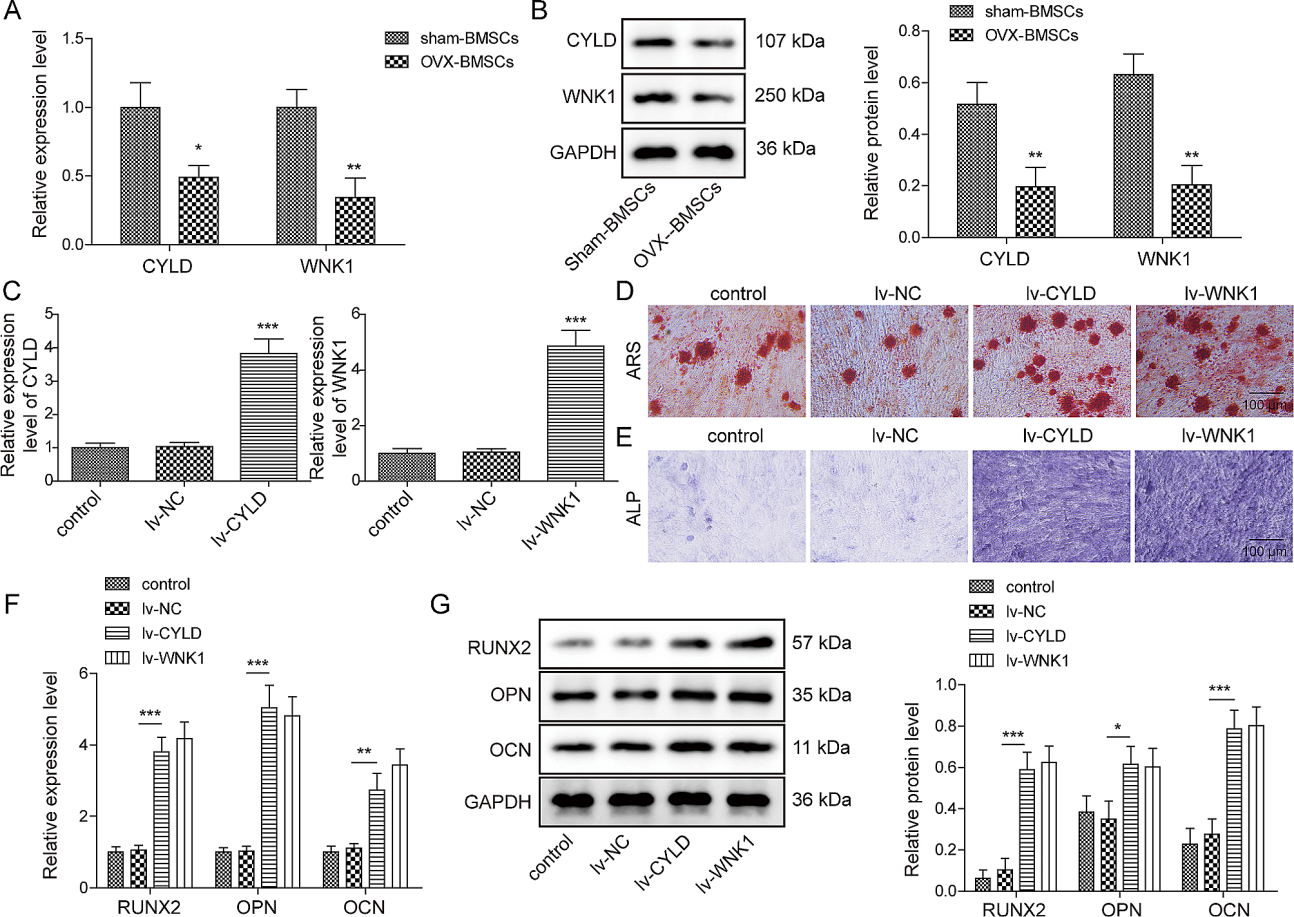
Overexpression of CYLD or WNK1 promoted osteogenic differentiation of OVX-BMSCs BMSCs were isolated from sham mice and OVX mice, and named as sham-BMSCs and OVX-BMSCs, respectively. **(A-B)** qRT-PCR and Western blot was used to evaluate CYLD and WNK1 expression in sham-BMSCs and OVX-BMSCs. **(C)** qRT-PCR was utilized to test the transfection efficiency of CYLD or WNK1 overexpression vectors in OVX-BMSCs. **(D-E)** ARS and ALP staining was performed to detect osteogenic differentiation of OVX-BMSCs after CYLD or WNK1 overexpression. **(F-G)** qRT-PCR and Western blot was conducted to measure the levels of osteogenesis-related markers in cells with CYLD or WNK1 overexpression. Data were represented as mean ± SD. *P < 0.05, **P < 0.01, ***P < 0.001

To further examine the impact of CYLD and WNK1 on osteogenic differentiation, we isolated BMSCs from the sham mice and OVX mice. Then, the results of qRT-PCR and western blot revealed that CYLD and WNK1 levels in OVX-BMSCs were extremely lower than that in sham-BMSCs (Fig. 2A-B). Next, we overexpressed CYLD and WNK1 by transfecting OVX-BMSCs with CYLD and WNK1 overexpressing vectors which packaged by lentivirus. Evidenced by qRT-PCR analysis, after transfection with the corresponding lentivirus overexpression plasmids, the expression of CYLD and WNK1 was markedly elevated (Fig. 2C). Alizarin red and ALP staining results revealed that overexpression of CYLD or WNK1 remarkably enhanced the alizarin red staining degree and ALP activity of BMSCs (Fig. 2D-E). Furthermore, after upregulation of CYLD and WNK1, the expressions of osteogenesis-related markers (RUNX2, OCN and OPN) were all obviously increased (Fig. 2F-G). Collectively, these data concluded that overexpression of CYLD or WNK1 promoted osteogenic differentiation of OVX-BMSCs.

### Upregulation of CYLD or WNK1 suppressed the formation of NLRP3 inflammasome in OVX-BMSCs

**Fig. 3:**
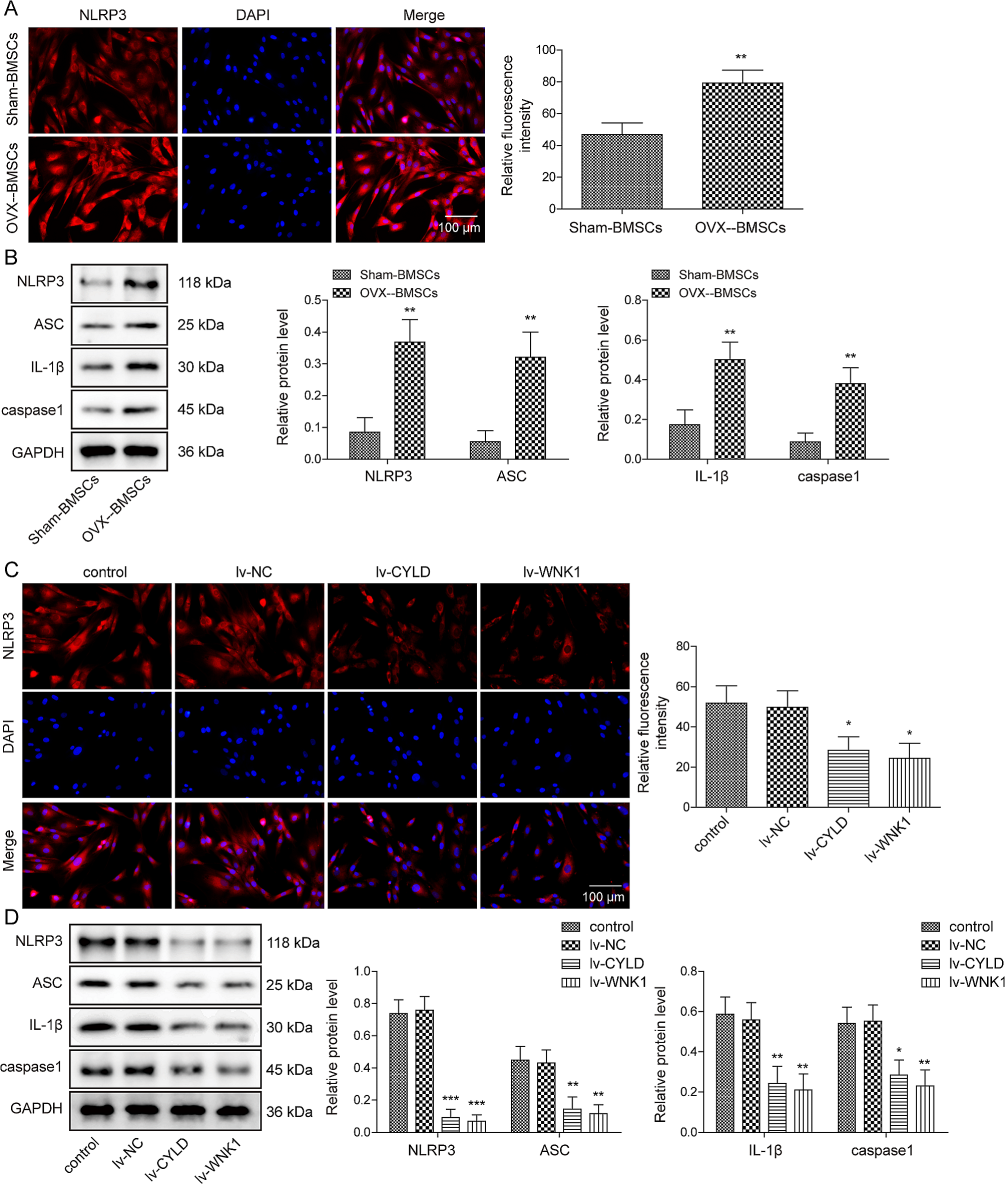
Upregulation of CYLD or WNK1 suppressed the formation of NLRP3 inflammasome in OVX-BMSCs (**A**) Immunofluorescence detection of NLRP3. (**B**) Western blot was used to analyze NLRP3, ASC, IL-1β and caspase 1 expression. (**C**) Immunofluorescence detection of NLRP3. (**D**) Western blot was performed to analyze NLRP3, ASC, IL-1β and caspase 1 expression. Results expressed as mean ± SD. *P < 0.05, **P < 0.01, ***P < 0.001

Subsequently, the levels of NLRP3 inflammasome in sham-BMSCs and OVX-BMSCs were detected using immunofluorescence staining and western blot. As suggested in Fig. 3A-B, the levels of NLRP3, ASC, IL-1β, and caspase 1 in OVX-BMSCs were extremely higher than that in sham-BMSCs. Next, the effect of CYLD and WNK1 on NLRP3 inflammasome formation was investigated. The results obtained showed that overexpression of CYLD or WNK1 significantly decreased NLRP3, ASC, IL-1β, and caspase 1 levels, compared to the control OVX-BMSCs (Fig. 3C-D). To sum up, CYLD or WNK1 overexpression effectively suppressed the formation of NLRP3 inflammasome.

### CYLD enhanced the protein stability by deubiquitination of WNK1

**Fig. 4:**
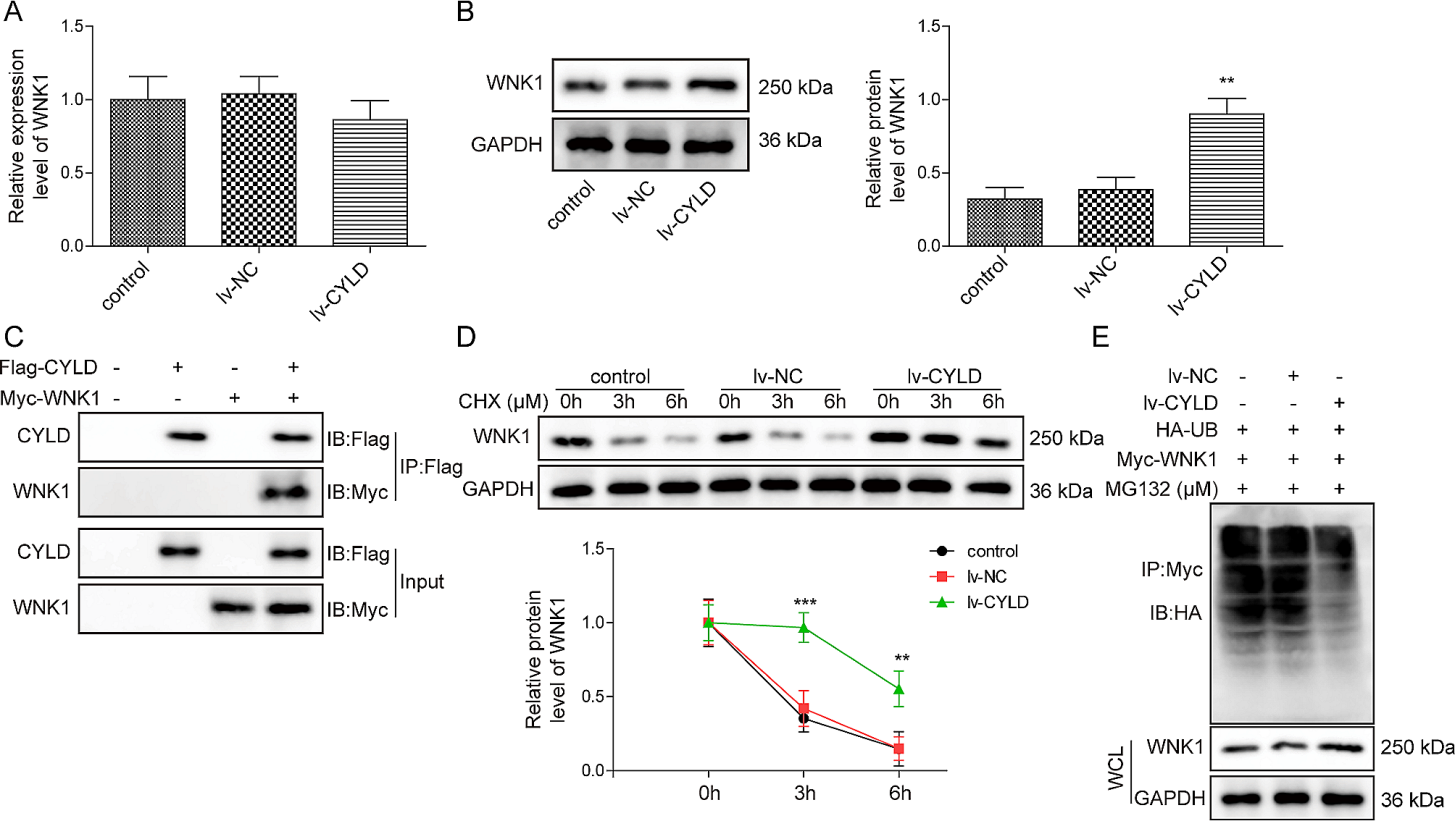
CYLD enhanced the protein stability by deubiquitination of WNK1 **(A-B)** Expression of WNK1 in CYLD overexpressed OVX-BMSCs was assessed by qRT-PCR and western blot. **(C)** Co-IP validated the binding relationship between CYLD and WNK1 in 293T cells. **(D)** OVX-BMSCs transfected with NC or CYLD overexpression vectors were incubated with 50 ng/ml CHX for 0, 6, 12 h. Then, western blot was adopted to measure WNK1 level. **(E)** In vitro detection of WNK1 protein ubiquitination. Values were expressed as mean ± SD. *P < 0.05, **P < 0.01, ***P < 0.001

Subsequently, we sought to explore whether CYLD and WNK1 in OVX-BMSCs exerted their effects through specific molecular mechanisms. Interestingly, we found that overexpressed CYLD had no significant effect on the mRNA expression of WNK1, but greatly increased the protein level of WNK1 (Fig. 4A-B). Next, Co-IP assay verified that there was a binding relationship between CYLD and WNK1 in 293T cells (Fig. 4C). Meanwhile, the endogenous Co-IP experiments in OVX BMSCs and normal BMSCs also confirmed CYLD and WNK1 interacted with each other (Supplementary Fig. 1B). Additionally, CHX treatment greatly accelerated the degradation of WNK1, whereas this trend was reversed by CYLD overexpression (Fig. 4D). Subsequently, the in vitro ubiquitination experiment further showed that CYLD upregulation dramatically inhibited the ubiquitination level of WNK1 (Fig. 4E). Thus, CYLD stabilized WNK1 by suppressing the ubiquitination of WNK1 in OVX-BMSCs.

#### Inhibition of WNK1 abolished the biological functions of CYLD overexpression in OVX-BMSCs

**Fig. 5:**
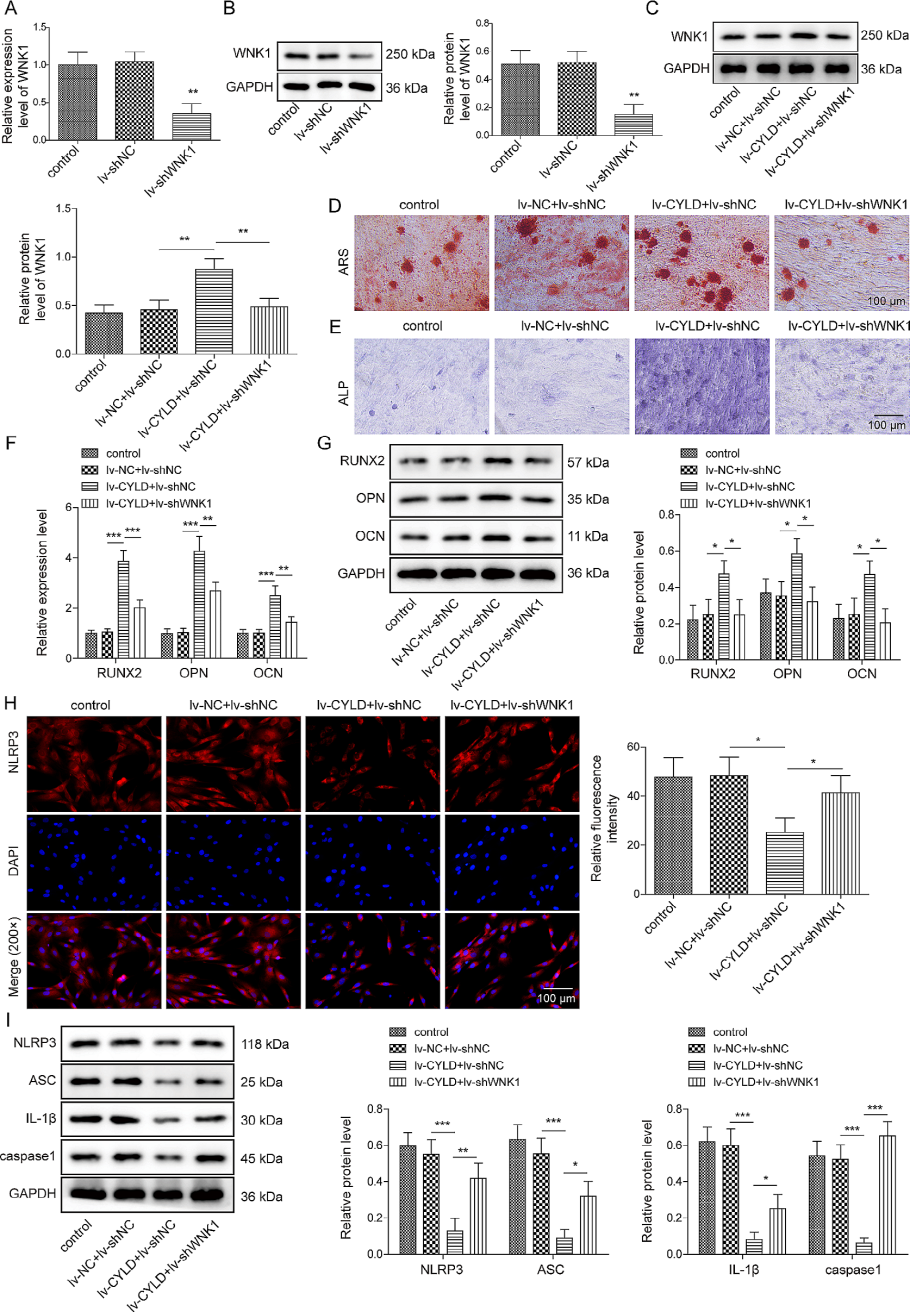
Inhibition of WNK1 abolished the biological functions of CYLD overexpression in OVX-BMSCs **(A-B)** OVX-BMSCs were transfected with lv-shNC or lv-shWNK1, then, qRT-PCR and Western blot were used to test WNK1 level. OVX-BMSCs were transfected with lv-CYLD or lv-CYLD + lv-sh-WNK1. **(C)** Western blot was performed to measure WNK1 expression. **(D-E)** ARS and ALP staining were utilized to detect osteogenic differentiation of OVX-BMSCs **(F-G)** qRT-PCR and Western blot were adopted to measure the levels of osteogenesis-related markers. **(H)** Immunofluorescence detection of NLRP3 expression. **(I)** Western blot was conducted to analyze NLRP3, ASC, IL-1β and caspase 1 levels. Error bars represent SD of the mean. *P < 0.05, **P < 0.01, ***P < 0.001

We proceeded to investigate whether the regulation of osteogenic differentiation by CYLD involved the modulation of WNK1. Based on the principle of rescue experiments, we knocked down WNK1 in CYLD overexpressing OVX-BMSCs. Firstly, the knockdown efficiency of lentiviral packaging sh-WNK1 was examined. As confirmed in Fig. 5A-C, sh-WNK1 transfection remarkably reduced the level of WNK1, and also weakened the promoting effect of CYLD overexpression on WNK1 expression. Then, Alizarin red and ALP staining assays uncovered that CYLD overexpression significantly increased the degree of alizarin red staining and ALP activity, but these effects were reversed upon silencing of WNK1 (Fig. 5D-E). Moreover, BMSCs stimulated by CYLD overexpression clearly elevated RUNX2, OPN, and OCN levels, whereas these impacts were dramatically weakened by WNK1 knockdown (Fig. 5F-G). Furthermore, the levels of NLRP3, ASC, IL-1β and caspase 1 were decreased by overexpressed CYLD, and these trends were also reversed by WNK1 silencing (Fig. 5H-I). Taken together, these findings revealed that CYLD could facilitate osteogenic differentiation of OVX-BMSCs through WNK1.

## Discussion

The primary clinical manifestation of OP is the reduction in bone mass level, resulting in decreased bone mineral density. This decrease in bone mass increases the risk of fractures among individuals with OP [33]. Following menopause in women and after the age of 50 in men, there is an imbalance between bone resorption and bone formation, with resorption surpassing formation. This imbalance leads to a decline in bone mass and heightened bone fragility, ultimately contributing to the onset and progression of OP [34, 35]. NLRP3 inflammasome is a cytosolic multiprotein complex composed of innate immune receptor protein NLRP3, adapter protein ASC, and inflammatory protease caspase-1, which plays a key role in host defense by promoting IL-1β and IL-18 releasing, and dysregulation of inflammasome activity is associated with the development of autoinflammatory diseases [36]. Besides, the formation of NLRP3 inflammasome is responsible for the progression of OP and osteogenic differentiation of BMSCs [37]. For example, Melatonin suppressed OP triggered by estrogen deficiency by inactivating NLRP3 inflammasome [31]. Irisin inhibited the NLRP3 inflammasome, thereby reducing the incidence of postmenopausal OP [38]. Additionally, the activation of NLRP3 inflammasome repressed the osteogenic differentiation of mesenchymal stem cells [39]. In the present work, we found that the expression of CYLD and WNK1 was downregulated, and NLRP3 inflammation was activated in OP mice, suggesting that CYLD and WNK1 may be involved in the formation of NLRP3 inflammasome in OP.

CYLD protein is a deubiquitinating enzyme recently identified, which can deubiquitinate various signaling molecules and play an important regulatory role in the activation of signaling in a variety of solid tumors [40]. CYLD also plays a regulatory role in a wide range of physiological processes, including immune response, inflammation, cell cycle progression, spermatogenesis, and osteoclastogenesis [15]. Additionally, CYLD is intricately linked to the development of OP. For instance, the recruitment of CYLD in response to iron oxide nanoparticles has been observed to suppress osteoclastogenesis and mitigate ovariectomy-induced bone loss [17]. Our present study showed that CYLD expression was decreased in bone tissues of OP mice and OVX-BMSCs. Furthermore, osteogenic differentiation of BMSCs could be positively regulated by CYLD. Literature has revealed that CYLD plays a crucial role in the regulation of inflammatory process. For instance, CYLD downregulation has been shown to enhance the pro-inflammatory effects of rheumatoid arthritis fibroblast-like synoviocytes by activating the NF-κB signaling [41]. CYLD prevented excessive IL-18 production in the colonic mucosa by deubiquitinating the NLRP6 inflammasome [42]. Similarly, we found that CYLD could reduce the formation of NLRP3 inflammasome in OVX-BMSCs. Therefore, our results imply that CYLD is an important factor in the osteogenic differentiation of BMSCs and OP progression.

WNK1 plays a vital role in maintaining ion homeostasis by regulating the trafficking or activity of various membrane transport proteins [43]. Multiple studies have highlighted the contribution of WNK1 in regulating blood pressure and electrolyte balance through its modulation of multiple ion channels and ion transporters [44]. Recently, low expression of WNK1 has been observed in OP patients [23]. In accordance with this, we identified WNK1 was decreased in OVX mice. Importantly, the osteogenic differentiation of BMSCs in OP mice was improved by WNK1 overexpression. It was reported that WNK1 regulated chloride sensing and inhibited the NLRP3 inflammasome [30]. Consistent with these findings, our data further suggested that WNK1 could suppress the formation of NLRP3 inflammasome in OVX-BMSCs. Altogether, our findings provide evidence that WNK1 induces osteogenic differentiation in OVX-BMSCs.

Deubiquitination is a significant type of protein post-translational modification that influences the localization, stability and function of target proteins within cells. This process also impacts various cellular physiological activities, including cell cycle regulation, differentiation, and apoptosis [45]. Based on the deubiquitination function of CYLD and bioinformatics analysis, we verified that CYLD, as a deubiquitinating enzyme, negatively regulated the activation of NLRP3 inflammasome by catalyzing the deubiquitination of WNK1 and increasing its protein stability. Similarly, Yang et al. reported that CYLD suppressed NEK7-mediated activation of NLRP3 inflammasome [46]. Moreover, WNK1 was found to repress NLRP3 activation by balancing intracellular Cl^−^ and K^+^ concentrations [30]. Therefore, it can be inferred that CYLD may inactivate the NLRP3 inflammasome by modulating WNK1. As we demonstrated, functional experiments confirmed that WNK1 silencing reversed the promotion of osteogenic differentiation and the inhibition of NLRP3 inflammasome formation by CYLD overexpression. Taken together, these findings suggested that CYLD inhibited the activation of NLRP3 inflammasome through WNK1 in OVX-BMSCs.

In summary, our study elucidated that CYLD induced osteogenic differentiation by deubiquitinating WNK1 to inhibit NLRP3 inflammasome formation. Our findings identify CYLD as a promising therapeutic target for OP. In future studies, we will continue to investigate the correlation between CYLD and WNK1, as well as unravel the precise mechanisms underlying this association in clinical OP patients.

### Electronic supplementary material

Below is the link to the electronic supplementary material.

Supplementary Material 1

## Data Availability

Data sharing not applicable to this article as no datasets were generated or analysed during the current study.

## Abbreviations

ARS: Alizarin red staining
ALP: Alkaline phosphatase
BMSCs: Bone marrow mesenchymal stem cells
BMD: Bone mineral density
BTMs: Bone turnover markers
BV/TV: Bone volume/trabecular volume
Co-IP: Co-immunoprecipitation
cDNA: Complementary DNA
CHX: Cycloheximide
CYLD: Cylindromatosis
DUBs: Deubiquitinases
DAB: Diaminobenzidine
HE staining: Hematoxylin and eosin staining
HRP: Horseradish peroxidase
IHC: Immunohistochemical staining
IL-1β: Interleukin-1β
NLRP3: NOD-like receptor family pyrin domain containing protein 3
OP: Osteoporosis
OVX: Ovariectomized
TB. N: Trabecular number
TB. Th: Trabecular thickness
WNK1: With no lysine [K] kinase 1
