# Supplementary figures and images for: CYLD alleviates NLRP3 inflammasome-mediated pyroptosis in osteoporosis by deubiquitinating WNK1
Source: J Orthop Surg Res. 2024 Apr 1;19:212. doi: 10.1186/s13018-024-04675-2 (PMC10983667; doi:10.1186/s13018-024-04675-2)

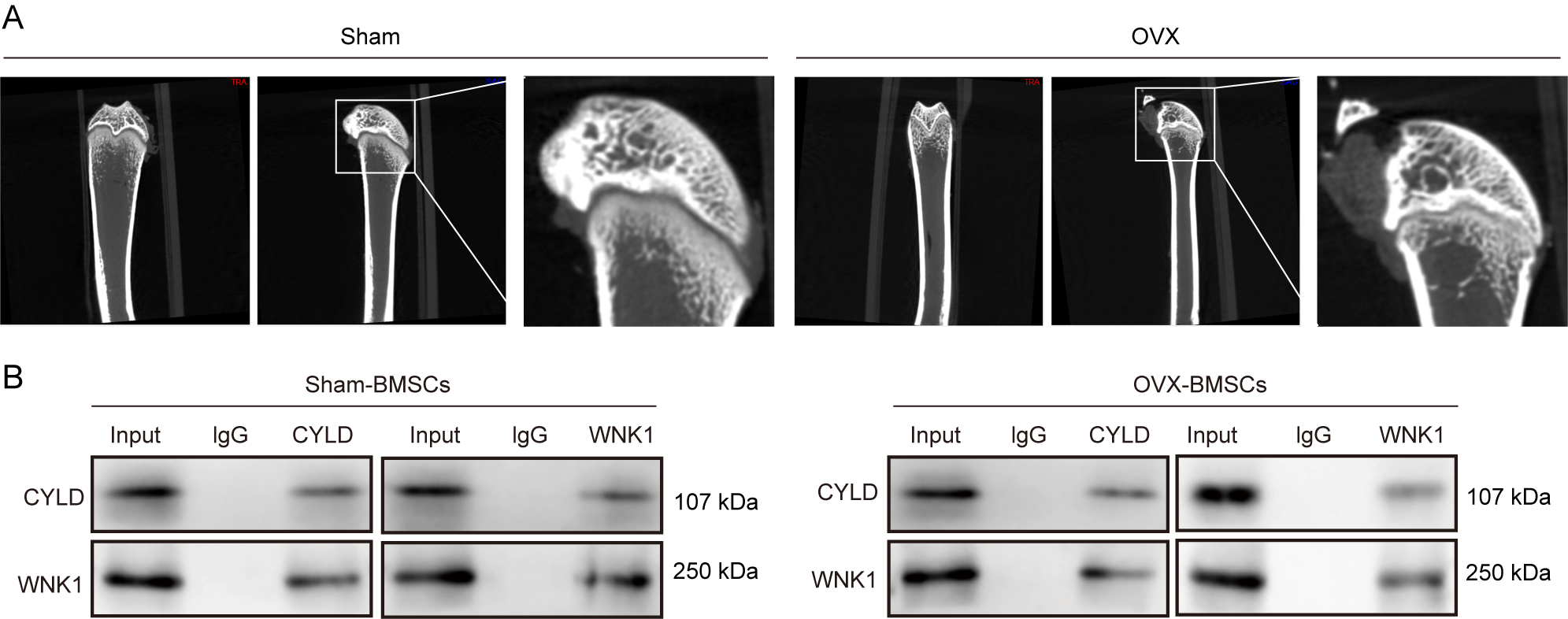

Supplement: Supplementary file 1 — Supplementary Material 1 [file 13018_2024_4675_MOESM1_ESM.png]
